# Management of Functional Pancreatic Neuroendocrine Neoplasms

**DOI:** 10.1007/s11864-023-01085-0

**Published:** 2023-04-27

**Authors:** Ludovica Magi, Matteo Marasco, Maria Rinzivillo, Antongiulio Faggiano, Francesco Panzuto

**Affiliations:** 1 Digestive Disease Unit, Sant’Andrea University Hospital, ENETS Center of Excellence, Via di Grottarossa 1035, 00189 Rome, Italy; 2 Endocrinology Unit, Sant’Andrea University Hospital, ENETS Center of Excellence, Rome, Italy; 3 Department of Clinical and Molecular Medicine, Sapienza University of Rome, Via Di Grottarrossa 1035, 00189 Rome, Italy; 4 Department of Medical-Surgical Sciences and Translational Medicine, Sapienza University of Rome, Via di Grottarossa 1035, 00189 Rome, Italy

**Keywords:** Neuroendocrine tumors, Insulinoma, Gastrinoma, Treatment, Somatostatin analogs

## Abstract

Functional pancreatic neuroendocrine neoplasms (pNENs) are rare and heterogeneous diseases in terms of both clinical and pathological aspects. These tumors secrete hormones or peptides, which may cause a wide variety of symptoms related to a clinical syndrome. The management of functional pNENs is still challenging for clinicians due to the need to control both tumor growth and specific symptoms. Surgery remains the cornerstone in the management of local disease because it can definitively cure the patient. However, when the disease is not resectable, a broad spectrum of therapeutic options, including locoregional therapy, somatostatin analogs (SSAs), targeted therapies, peptide-receptor radionuclide therapy (PRRT), and chemotherapy, are available. The present review summarizes the main key issues regarding the clinical management of these tumors, providing a specific highlight on their therapeutic approach.

## Introduction

Pancreatic neuroendocrine neoplasms (pNENs) are heterogeneous diseases arising from the neuroendocrine system of the pancreas. Although considered relatively rare, their incidence has been increasing over time, representing the second leading cause of pancreatic cancer [1]. Pancreatic NENs are clinically divided into nonfunctional (70%) and functional (30%) tumors according to their ability to secrete hormones or peptides, which cause a wide variety of symptoms related to a clinical syndrome [2, 3]. Up to sixteen types of functional pNEN syndromes have been described, but the two most common are gastrinomas and insulinomas [3, 4]. Pancreatic NENs may be sporadic or arise in the context of an inherited syndrome such as multiple endocrine neoplasia type 1 (MEN1) or type 4 (MEN4), von Hippel‒Lindau (VHL) disease, neurofibromatosis type I (NF1), and tuberous sclerosis [5]. In the hereditary setting, pNENs are usually multifocal, the onset of disease is earlier, and other endocrine disorders or malignancies are present [2]. Hereditary pNENs are usually well differentiated and are associated with hormone secretion and functional endocrine syndromes more frequently than sporadic pNENs [6].

Although the prognosis of these tumors is affected by several factors, including tumor size, staging, and grading, proliferative activity, expressed as the Ki-67 index, is considered the strongest prognostic factor [7]. Based on the proliferative activity and tumor differentiation, tumors may be classified into well-differentiated NENs (NET G1: Ki-67 < 3%, NET G2: Ki-67: 3–20%, and NET G3: Ki-67 > 20%) and poorly differentiated NEC G3 [8].

The clinical presentation of functional pNENs is heterogeneous depending on the specific kind of hormonal oversecretion causing a wide variety of symptoms.

Most common functional pNENs include insulinomas, characterized by severe hypoglycemia with a usually low risk of malignant behavior (5% of cases), and gastrinomas caused by hypersecretion of gastrin which is responsible for a clinical syndrome known as Zollinger-Ellison syndrome (ZES) characterized by gastroesophageal reflux disease (GERD), peptic ulcer disease (PUD), and diarrhea with a good response to proton pomp inhibitors (PPI) therapy [3, 9–11]. In addition, other generic symptoms related to the tumoral mass effect, such as nausea, vomiting, pain, jaundice, and anorexia, may be present.

Once the clinical diagnosis of functional pNEN is proposed, the tumor needs to be localized and staged using a combination of conventional radiological imaging (CT scan, MRI), endoscopy ultrasonography (EUS), and functional imaging (68Ga DOTATOC PET/CT) [3, 10].

The presence of a functional syndrome makes the handling of these uncommon types of cancers more difficult: clinicians must both manage tumor growth and hormonal hypersecretion. Thus, these neoplasms should be referred early to dedicated NEN centers and approached by a multidisciplinary team [12].

In this review, we focused on the clinical management of functional pNENs, highlighting the two clinical aspects to consider in these patients: the tumor disease itself and the clinical aspects related to the overexpressed hormone.

## Clinical aspects of functional p-NENs

The clinical presentation of functional pNENs varies according to the hormone oversecreted by the neoplasm. Since the management of these tumors really depends on the clinical syndrome related to each tumor, in this section we highlight the main clinical aspects of the most common functional pNENs (Table 1).

**Table 1:** Functional pancreatic endocrine neoplasms (pNEN) syndromes

Functional syndrome	Incidence (new cases/10^6^ population/year)	Hormone hypersecretion	Frequency of pancreatic origin	Clinical presentation	Diagnostic tests	Therapeutic options for symptom control
Insulinoma	1–3^3^	Insulin	> 99%	-Hypoglycemia (usually fasting) -Neuroglycopenic symptoms (confusion, seizures, blurred vision, coma) - Autonomics symptoms (diaphoresis, tremor, weakness)	-72-h fasting test - Plasmatic insulin and c-peptide - Plasmatic pro- insulin	-Diazoxide -Lanreotide/octreotide -Everolimus -PRRT
Gastrinoma (Zolliger-Ellison syndrome)	1–1.5^11^	Gastrin	Pancreas (25%) Duodenum (70%) Adjacent tissues (5%)	-Peptic ulcer disease -Gastroesophageal reflux -Diarrhea -Abdominal pain -Weight loss	-Fasting serum gastrin (FSG) - Gastric pH measurement -Secretin test -Basal acid output	-PPIs -H2 antagonist -Lanreotide/octreotide -PRRT
Glucagonoma	0.01–0.1^3^	Glucagon	100%	-Diabetes mellitus -Neuropsychiatric symptoms (depression, dementia, ataxia) -Necrolytic migratory erythema (NME) -Deep thrombosis	Fasting plasma glucagon (associated with typical sign and symptoms of glucagonoma)	-Lanreotide/octreotide -PRRT
VIPoma	00.5–0.2^3^	Vasoactive intestinal peptide	90%	-Watery secretory diarrhea (up to 3000 ml per day) -Symptoms related to dehydration and hypokalemia (nausea, vomiting, lethargy, cramps) -Flushing (8–20%)	-VIP serum level dosage -Fecal osmotic gap evaluation	-Lanreotide / octreotide -Sunitinib -Pasireotide
Somatostatinoma	Extremely rare	Somatostatin	Pancreas (56%) Duodenum/jejunum (44%)	-Diabetes mellitus (60%) Cholelithiasis (70%) -Steatorrhea -Vomiting -Nausea -Abdominal pain	-Fasting serum somatostatin -24-h urine level of 5-HIAA	-Lanreotide / octreotide -PRRT -Pasireotide
Ectopic acromegaly	Unknown	GH/GHRH	30%	-Acromegaly	GH suppression test	-Lanreotide/octreotide -PRRT -Pasireoitide
Ectopic Cushing’s syndrome	Extremely rare	ACTH/CRH	4–16%	-Cushingoid habitus -Diabetes mellitus -Arterial hypertension -Hypokalemia -Osteoporosis/vertebral fractures -Sepsis	-Serum ACTH and cortisol concentrations -24-h urinary free cortisol concentration -High-dose dexamethasone suppression test	-Ketoconazole -Metyrapone -Osilodrostat -Mitotane -Lanreotide/octreotide -PRRT -Pasireotide -Bilateral adrenalectomy

*PRRT* peptide radio receptor therapy, *VIP* vasoactive intestinal peptide, *5-HIAA* 5-hydroxy indole acetic acid, *GH* growth hormone, *GHRH* growth hormone releasing hormone, *ACTH* adrenocorticotropic hormone, *CRH* corticotropin-releasing hormone

### Insulinoma

Insulinoma is the most common functional pNEN with an incidence of 1–3 per million population/year, usually presents as a small solitary tumor with a low risk of malignant behavior (5% of cases) [3]. It is characterized by the hypersecretion of insulin, which causes severe hypoglycemia with possible neurological symptoms, such as seizures, confusion, blurred vision, and coma. Furthermore, the activation of the sympathoadrenal system is responsible of autonomics symptoms including palpitations, diaphoresis, tremors, and weakness [13]. Insulinoma should be suspected in the presence of the classical Whipple’s triad: symptoms of hypoglycemia, documented low plasma glucose concentration, and relief of symptoms after glucose administration [9]*.* The gold standard for the diagnosis is a 72-h fast; values of insulin equal to or greater than 3 μU/ml in the presence of a blood glucose less than 3 mmol/l (55 mg/dl) are highly suggestive, but the diagnosis is established in case of blood glucose less or equal than 2.2 mmol/l (40 mg/dl) and values of insulin equal to or greater than 6 μU/ml. In addition, the determination of C-peptide and pro-insulin in the plasma is recommended, particularly for those patients with low or undetectable insulin levels in the blood [3, 13].

### Gastrinoma

Gastrinoma is the second most frequent functional tumor of the pancreas, with an incidence of 1–1.5 cases/million per year [11]. Only 25% of gastrinomas are in the pancreas, whereas 70% are found in the duodenum and 5% in the adjacent tissues. The gastrin, which is the hormone overexpressed by this tumor, is responsible for a clinical syndrome known as Zollinger-Ellison syndrome (ZES) characterized by gastroesophageal reflux disease (GERD), peptic ulcer disease (PUD), and diarrhea with good response to proton pomp inhibitors (PPI) therapy [11]. Due to these common and non-specific gastrointestinal symptoms, the diagnosis of ZES is often delayed. However, the presence of a recurrent PUD in the absence of *Helicobacter* *pylori* infection, the association with a chronic diarrhea, and a prompt response to PPI therapy should be suspicious for a ZES diagnosis [3, 11, 13]. Furthermore, it is important to remark that about 25% of patients with gastrinoma occur in the context of MEN-1 which is characterized by parathyroid adenomas, pituitary, and pancreatic tumors [3]. The diagnosis of gastrinoma requires the demonstration of hypergastrinemia associated with gastric hyperchlorhydria. Notably, the combination of fasting serum gastrin (FSG)  > 10 times elevated in the presence of a gastric pH below 2 is considered diagnostic; however, a proportion of patients with ZES (60%) have a gastric pH below 2 but a FSG  < tenfold elevated. In this setting, other causes of hypergastrinemia (e.g., PPI, atrophic gastritis, chronic renal failure, *Helicobacter* *pylori* infection, extensive small bowel resection) must be excluded and additional tests such as secretin test and basal acid output must be performed [14]. However, some specific tests, including esophageal pH-recording and secretin test, are not widely available in clinical practice. Another difficult aspect in the diagnosis of gastrinoma is that the primary tumor is not always detectable on conventional imaging, especially in MEN-1 patients with small multifocal lesions [3, 4, 10, 11].

Thus, we suggest that in case of symptoms highly suspicious for ZES, once other common causes of hypergastrinemia are excluded, an accurate imaging study such as endoscopic ultrasonography (EUS) to detect the primary tumor may be proposed.

### Glucagonoma

Glucagonomas are rare pancreatic NENs with an incidence of 0.01–0.1/million per year [3]. They are usually large solitary lesions, all occurring in the pancreas, mainly in the tail or in the body, where alpha cells are more represented. More than 60% are malignant, and over 50% are metastatic at time of diagnosis [13]. Glucagonoma syndrome is caused by the excess of glucagon produced by the neoplasms, and it is clinically characterized by diabetes mellitus, weight loss, deep vein thrombosis, neuropsychiatric symptoms (depression, dementia, ataxia), and a particular dermatosis known as necrolytic migratory erythema (NME) that presents in almost 90% of patients. NME is characterized by erythematous papules or plaques which gradually enlarge and coalesce to form bullous lesions. Also, the mucous membranes may be involved causing angular cheilitis, glossitis, stomatitis, and blepharitis [3, 13, 15]. The diagnosis of glucagonoma is established by the combination of fasting plasma glucagon  > 500 pg/ml, clinical symptoms related to glucagonoma syndrome (especially NME), and abnormal imaging [13]_._

### VIPoma

VIPoma is a rare functional pNEN and usually presents as a sporadic solitary tumor greater than 3 cm in diameter and typically located in the tail of the pancreas (75%) [13]. VIPoma is characterized by the excessive secretion of the vasoactive intestinal peptide (VIP), a neurotransmitter of the central nervous system present in not only neurons of the gastrointestinal tract, liver, and pancreas but also the lungs and adrenal glands. In the gastrointestinal tract, VIP exerts its activity through the stimulation of the pancreatic and intestinal secretions, the contraction of enteric smooth muscle cells, and increasing the gastrointestinal blood flow [16, 17].

Thus, the oversecretion of VIP causes severe watery diarrhea, which may result in hypokalemia, dehydration, achlorhydria, and metabolic acidosis. The stool volume may exceed 3000 ml/day in up to 70–80% of patients even during fasting (secretory diarrhea). Other symptoms include flushing (15–30%), hypotension, hypercalcemia (25–50%), and hyperglycemia (20–50%). In addition, electrolyte imbalances may result in lethargy, nausea, vomiting, muscle cramps, and weakness [13, 16, 17]. The diagnosis is made in the presence of a large volume of secretory watery diarrhea, evidence of a tumor mass on imaging, and VIP levels greater than 500 pg/ml [13].

### Other rare functional pancreatic NEN

Other rare functional pNENs include somatostatinoma, tumors associated with ectopic Cushing syndrome, carcinoid syndrome, and hypercalcemia syndrome (PTHrP-secreting tumor) [3] the characteristics of which are summarized in Table 1. These tumors represent less than 10% of pNENs and are usually within the pancreas with exception of somatostatinoma which is found in the duodenum/jejunum in 44% of patients [18]. They clinically present with symptoms related to the hormone overexpressed and a proportion of these patients may develop a second syndrome during the disease course [3, 13].

## Therapeutic approach in functional p-NENs

**Fig. 1:**
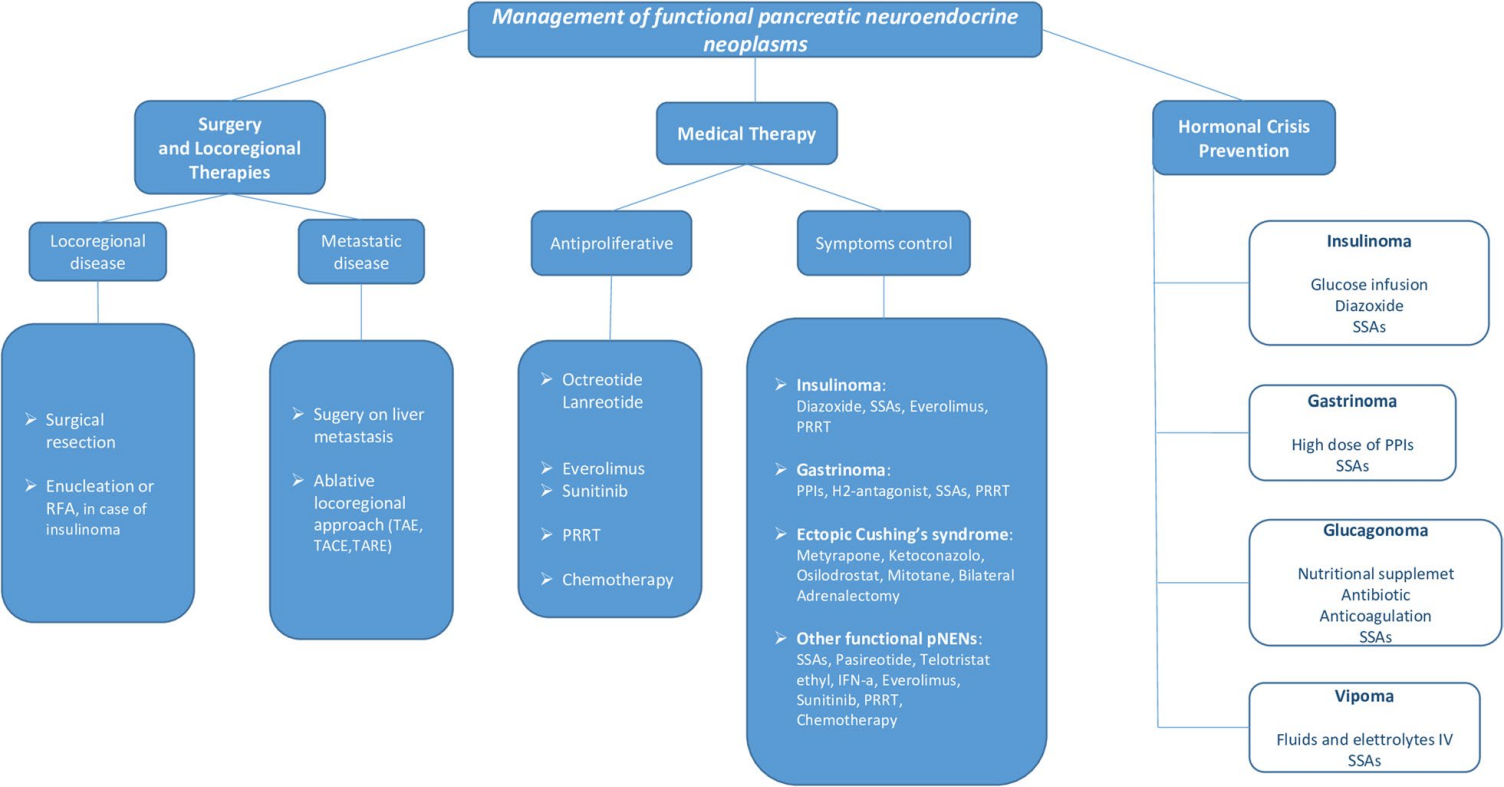
Management of functional pancreatic neuroendocrine neoplasms. This figure represents a practical algorithm for the management of functional pNENs. Abbreviations: RFA, radiofrequency ablation; MEN 1, multiple endocrine neoplasia syndrome type 1; TAE, transarterial embolization; TACE, transarterial chemoembolization; TARE, transarterial radioembolization; PRRT, peptide radio receptor therapy; SSAs, somatostatin analogs; PPIs, proton pump inhibitors; IFN-a, interferon alpha.

After the accurate diagnostic assessment and staging of disease, the management of functional pancreatic neuroendocrine tumors remains challenging for clinicians (Fig. 1).

### Management of local/locoregional disease

Surgery with radical intent represents the treatment of choice for locoregional or locally advanced functional G1 or G2 pNENs [2, 19]*.* However, before planning a surgical approach, tumor localization, tumor size, and local invasiveness need to be considered. In functional pNENs, surgery remains the cornerstone to achieve a cure and complete symptomatic relief. Standard pancreatectomy (distal or pancreaticoduodenectomy) with lymphadenectomy is the preferred approach; however, in cases of small benign insulinomas (<1 cm) localized in the head or tail of the pancreas, a more conservative approach by surgical enucleation should be considered [2, 10]. Pancreaticoduodenectomy or distal pancreatectomy may be necessary in cases of local recurrence after enucleation [20•].

In patients with gastrinomas located in the pancreas (approximately 25% of patients) and MEN1, the role of surgery remains debated, and a risk-benefit assessment should always be evaluated [2, 21, 22]. In this setting, gastrinomas are usually multifocal, and it is difficult to achieve a radical surgery. One study reported that only 16% of patients with MEN1 were free of disease after duodenectomy or pancreaticoduodenectomy whereas in more recent studies up to 77% were eugastrinemic after a surgical management [23]. Thus, in the setting of hereditary pancreatic gastrinomas, even if surgery of the primary tumor remains the only curative chance, the risks of a pancreatic surgery, especially when the lesion is in the head of the pancreas, the relatively indolent behavior of these tumors, the possibility of a multifocal localization, and the opportunity to control the syndrome with medical therapy, must be taken into account [2, 11, 23]*.* Unfortunately, other functional pancreatic NENs (glucagonoma, somatostatinoma, ectopic Cushing’s syndrome, etc.) are usually metastatic at the time of initial diagnosis; thus, surgical options should be considered only for debulking or symptom relief.

When an ectopic Cushing’s syndrome occurs in patients with resectable tumors, the radical surgery could be performed upfront or postponed after normalization of cortisol with medical therapy. This option should be preferred in cases of severe hypercortisolism [24].

For patients who are not eligible for surgery, EUS radiofrequency treatment represents a valid alternative to induce necrosis of the pNEN and stop hormonal hypersecretion, particularly if the tumor is smaller than 2 cm and hormone-related symptoms are present. Before planning EUS radiofrequency treatment, the distance between the lesion and pancreatic main duct must be considered [25]. Some studies have reported a complete response rate up to 95% with no additional procedure-related adverse events [26, 27••]. EUS-directed ablation using ethanol injection or CT-guided RFA appears to be a successful treatment for insulinomas in patients with sporadic disease or MEN1 [10].

### Metastatic disease

#### Surgery

In the setting of metastatic disease, surgery may play a role in both achieving symptom control and reducing the disease burden. However, before considering a surgical approach, some factors must be assessed: first, NEC G3 should be excluded because of the high risk of recurrence; metastases should be exclusive or predominant in the liver, the presence of extra-abdominal disease needs to be ruled out, and finally, the distribution and localization of liver metastases should be considered. Unfortunately, only 5–15% of patients with liver metastatic disease can be subjected to resection [2]. In patients with ectopic Cushing’s syndrome, a bilateral adrenalectomy could be taken into account to stop hypercortisolism-related symptoms. This condition could be life-threatening because of a high risk of thrombosis and cardiovascular events, hypokalemia, and sepsis. Bilateral adrenalectomy should be considered upfront in case of severe hypercortisolism, after medical therapy when not rapidly effective, or in case of toxicity or drug-drug interactions [28••].

Finally, in very selected cases, liver transplantation may be considered when a surgical approach is not feasible [2, 22, 29].

#### Locoregional therapy

In patients who are not eligible for transplantation or metastasis resection, an ablative locoregional approach (transarterial embolization (TAE), transarterial chemoembolization (TACE), radioembolization or selective internal radiotherapy (TARE), or radiofrequency ablation (RFA)) is recommended [29]. Since liver metastases from neuroendocrine tumors are highly vascularized by the hepatic artery, all these techniques are safe and effective. From the available data, TACE seems to be more effective than TAE in the setting of liver metastases from pancreatic NENs. However, both of these techniques improve symptoms and quality of life in 60–90% of patients. Future large prospective studies are needed to establish which treatment between TAE, TACE, or selective internal radiotherapy (SIRT) is the most effective one [30]. Finally, combining treatments (e.g., resection and RFA) may completely remove the tumor, but data on metastatic functional pNENs are scarce [2].

#### Antiproliferative therapy

Notably, somatostatin analogs (SSAs), including octreotide and lanreotide, represent the first-line therapy used to control both clinical symptoms and proliferative disease. SSAs are usually recommended for slowly progressive low proliferative pNENs [31]. This strategy is a long-term therapy with mild adverse effects that occur in approximately 50% of patients, including flatulence, diarrhea, nausea, gallstones, glucose intolerance, and exocrine pancreatic insufficiency [32, 33].

Among targeted agents, both everolimus and sunitinib are approved for advanced progressive pNENs. Everolimus is an oral m-TOR inhibitor whose role in pNENs has been well investigated in the phase 3 RADIANT-3 trial, showing an improvement in PFS (11.4 months in the everolimus arm vs. 5.4 months in the placebo arm), but no significant changes in OS were observed [34]. The side effects of everolimus include hyperglycemia, diarrhea, cytopenia, pneumonitis, and stomatitis [35]. Sunitinib (37.5 mg/day) is a tyrosine kinase inhibitor of the PDGF receptor and VEGF receptor subtype. The efficacy of this drug has been evaluated in a phase 3 trial, which demonstrated a significant improvement in PFS (11.4 months in the sunitinib arm vs. 5.5 months in the placebo arm) however with no significant OS benefit [36]. Sunitinib is approved for patients with advanced, progressive well-differentiated pNENs. Side effects associated with sunitinib include nausea, diarrhea, fatigue, cytopenia, thyroid dysfunction, heart failure, and arterial thromboembolism [2, 35].

Peptide radionuclide receptor therapy (PRRT) with 177-lutetium-labeled somatostatin analogs is a valid option for functional pNENs. Recently, PRRT was approved for all types of NENs expressing somatostatin receptors based on the NETTER-1 trial, showing antitumor efficacy in patients with advanced GEP NENs. Moreover, PRRT has demonstrated effective symptom control in refractory functional pancreatic syndromes [29, 35]. PRRT is usually considered a safe treatment; however, up to 3–4% of patients may develop myelotoxicity, including leukemia and bone marrow dysplasia [2]. Moreover, a final analysis of the phase 3 NETTER-1 trial showed that during long-term follow-up, 3 (3%) of 111 patients in the ^177^Lu-Dotatate group had serious adverse events, and 2 (2%) of these patients developed myelodysplastic syndrome, without any new cases of hematological events [37••]. Furthermore, preventing a hormonal crisis with dedicated pretreatments is important in the presence of a functional syndrome [38••]. According to the literature, gastrinomas seem to be one of the malignant pNENs that are most responsive to PRRT [11].

Finally, chemotherapy remains the treatment of choice for advanced progressive functional pNENs or in high-grade NEC G3. Many possible drug combinations are available, including streptozocin, doxorubicin, and fluorouracil [2]. Additionally, the combination of temozolomide and capecitabine has a role in this setting because it was associated with significantly improved PFS (22.7 months) and OS (38 months) compared to temozolomide alone [35].

#### Medical therapy for symptom control

##### Insulinoma

The first step to control symptoms related to insulinoma is dietary modification with small and frequent feedings, which may be the only strategy used before surgical intervention. A valid option to control hyperglycemic symptoms includes diazoxide, which is an inhibitor of insulin release that acts on ATP-sensitive potassium channels on insulinoma cells and is effective in approximately 50% of cases. Unfortunately, diazoxide is associated with some adverse effects: edema (because of fluid retention and generally diazoxide is used with a diuretic), hirsutism, thrombocytopenia, and renal failure [4].

SSAs control hypoglycemic symptoms in up to 50% of patients with nonmetastatic insulinomas [32]. However, their use requires careful monitoring because they may inhibit not only ectopic insulin secretion but also the release of counterregulatory hormones, such as glucagon, which can worsen hypoglycemia in some patients [4].

If the disease is localized, this approach usually involves a limited period prior to surgery. However, if the insulinoma is not susceptible to resection, other therapeutic options are employed in the management of tumor symptoms.

Even if everolimus is not registered for this indication by the EMA and FDA, it could be used for uncontrolled insulinoma symptoms due to its direct suppressive role in insulin secretion [2]. Also, PRRT may be employed in the management of refractory insulinomas. One study that evaluated the efficacy of PRRT in 34 patients with functional pNENs (14 insulinomas) showed a median PFS of 18.1 months with a concurrent increase in quality of life [38••]. However, an acute aggravation of symptoms, such as worsening hypoglycemia, might occur in some cases [2].

##### Gastrinoma

In patients with ZES, the main category of drugs to control acid hypersecretion is proton pump inhibitors (PPIs). Since the 1980s, the use of PPIs has lessened the need for surgery. In uncomplicated PUD associated with ZES, a dosage of omeprazole equivalent to 60 mg/day is usually sufficient. However, in patients with severe PUD and in the presence of MEN1 or prior Billroth 2 resection, the dosage could be higher, up to an omeprazole equivalent of 40–60 mg BID [32]. When mucosal healing is achieved, the dosage of PPI could be reduced to 20 mg BID omeprazole equivalent. Importantly, this type of patient requires long-term therapy with PPIs; thus, adverse events, such as deficits in vitamin B12 and magnesium, may be considered [4, 10]. In general, PPIs have significantly decreased the morbidity and mortality resulting from severe ulcer disease. In 60% of patients, ulcer healing occurs within 2 weeks; in 90–100% of patients, healing occurs within 4 weeks [10]. When PPIs cannot be administered, patients can receive histamine H2 receptor antagonists. SSAs are useful to reduce levels of gastrin and acid secretion in patients with ZES, but they are rarely administered for this indication [32]. Finally, recent studies underline the efficacy and safety of PRRT in patients with gastrinoma who are nonresponders to previous therapy [38••].

##### Ectopic Cushing’s syndrome

Some of these patients present with severe Cushing’s syndrome with symptoms that could be difficult to control under long-term surveillance, and a proportion of them may need bilateral adrenalectomy to definitely achieve remission of hypercortisolism and related symptomatology.

Medical therapy to restore eucortisolemia is represented by steroidogenesis inhibitors (ketoconazole, metyrapone, osilodrostat) and mitotane, an adrenolytic agent. These drugs need to be used cautiously because of the potential severe side effects and pharmacological interference with antiproliferative agents [4, 24]. Some cases with a significant response to SSAs have been reported, and these drugs are generally well tolerated. Besides octreotide and lanreotide, the panligand pasireotide and the D2-selective dopamine agonist cabergoline were reported to be potentially useful in ectopic Cushing’s syndrome [39, 40]. Finally, PRRT may be a valid option in malignant/refractory cases of Cushing’s syndrome of pNENs [38••].

##### Other functional pNENs

SSAs represent the treatment of choice to manage the symptoms of other functional pNENs (VIPoma, glucagonoma, somatostatinoma, and ectopic acromegaly), with partial symptomatic control in up to 70% of cases. Usually, these are drugs well tolerated, and they are administered monthly, e.g., 20–30 of mg/mo Octreotide or 60–120 of mg/mo Lanreotide Autogel. In case of uncontrolled symptoms, the SSA dose is commonly increased by shortening the injection interval to 3 or even 2 weeks [2].

Telotristat ethyl (oral inhibitor of tryptophan hydrolase) was recently registered for the treatment of patients with carcinoid syndrome, and it has demonstrated a significant improvement in diarrhea. It can be recommended for this indication as an add-on treatment to SSA [2]*.*

Interferon alpha (IFN-a) may be used for symptom control (3–5 million IU s.c. three times weekly), particularly in patients with VIPoma or rare forms of carcinoid syndrome related to pNENs; however, its administration is discouraged because of its broad spectrum of side effects [2, 41].

Finally, other treatment options available to control hormone excess in these rarer functional pancreatic syndromes include targeted therapy, PRRT, and chemotherapy. Sunitinib seems to be effective in reducing diarrhea in VIPoma, whereas PRRT may be a valid therapeutic option in cases of refractory syndromes [4]. Some chemotherapeutic regimens, such as streptozotocin and 5-fluorouracil, resulted in symptom control other than tumor growth proliferation in glucagonoma [23].

### Hormonal crisis prevention

Before planning an invasive treatment, such as surgery, locoregional therapy, and PRRT, it is mandatory to prevent a hormonal crisis due to the acute stress induced by the procedure with the release of high hormonal levels [41]. The prevention of hormonal crises should be considered in all patients, especially those with insulinoma, VIPoma, and gastrinoma.

In patients with gastrinoma, the administration of high-dose PPIs before and after procedures is mandatory because of predisposing gastrointestinal perforation and hemorrhage. A starting dose of 80 mg of pantoprazole administered via 15-min infusions every 8 h is recommended.

For patients with VIPoma, diarrhea inhibitors or, rarely, the IV administration of fluids and electrolytes should be prescribed to avoid severe loss of potassium and bicarbonate leading to metabolic acidosis. Patients with glucagonoma require SSA treatment, nutritional supplementation, and antibiotics to heal the skin lesion prior to surgery and perioperative anticoagulation (high-dose molecular heparin) to prevent thrombosis [42]. In patients with insulinoma treated with PRRT, a hormonal crisis may develop in up to 10% of patients; in this setting, the main goal is the prevention of hypoglycemia via glucose infusion or octreotide. Therapy needs to be initiated with dietary instructions, followed by a short-acting octreotide. Octreotide should be discontinued 24 h before PRRT, and it can then be restarted after the amino acid infusion [4, 38••]. During surgical procedure, infusion of glucose is discontinued to monitor glucose level, in particular the insulin/glucose ratio, because it seems relevant in assessing successful removal of an insulinoma. Instead, during the first period after surgery, in some cases, it could be necessary administration of small insulin doses. Otherwise, diazoxide is not always recommended before surgery because it can cause severe fluid retention [42].

## Prognosis of functional pNENs

The prognosis of pancreatic NENs is affected by several factors, including tumor size, staging, grading, and proliferative activity expressed as the Ki-67 index [7, 8]. Other prognostic factors include the presence of calcifications on CT scanning, the extent of liver metastases, the presence and number of lymph nodes involved, the absence of generic symptoms, and some molecular alterations (e.g., loss of DAXX or ATRX) [10]. Moreover, the behavior of functional pancreatic NENs is different for each tumor. Insulinomas are usually benign neoplasms (90–95%) and 95% of these can be surgically resected with successful surgical removal in almost all patients. However, in the presence of lymph node metastases, the prognosis is less than 24 months [3, 43•].

Almost 25% of patients with gastrinoma are metastatic at the time of their initial diagnosis, with a worse prognosis if compared with non-metastatic patients; surgical resection of the primary tumor is in fact associated with a survival rate  > 80% at 15 years [11]. Other factors associated with a worse prognosis include uncontrolled gastric acid hypersecretion, female gender, tumor size, absence of MEN1, development of ectopic Cushing’s syndrome, and bone metastases [3]. In patients with gastrinoma and MEN1, the prognosis seems to be better compared to patients with sporadic tumors particularly for those patients with lesions less than 2.5 cm [23]. Thus, as mentioned above, the decision for surgical management in MEN 1 gastrinomas should always be carefully discussed in a multidisciplinary team. Conversely, the other functional pancreatic NENs are usually metastatic at the time of initial diagnosis, with a worse prognosis and a 5-year survival ranging from 29 to 45% [3].

Finally, the prognostic significance of other functional status in pancreatic NENs is controversial. Non- functional pNENs have been generally considered to have a worse prognosis, probably due to the delayed diagnosis in the absence of specific tumor symptoms. However, the recent improved use of radiology imaging resulted in an increasing diagnosis of incidental asymptomatic small pNENs discovered at an earlier disease stage; thus, the prognostic role of hormone hypersecretion on pNEN survival remains unclear, particularly for non-metastatic disease [44]. In fact, it is important to remark that, with the exception of insulinomas, most patients with functional pNENs are metastatic at initial diagnosis, showing malignant behavior [45].

## Conclusions

Approaching functional pNENs remains challenging for physicians because both symptoms and tumor growth need to be simultaneously controlled. Surgery remains the only therapy able to provide a definitive cure for patients with limited disease. In patients not suitable for surgery due to unresectable disease, several systemic therapies are available, including somatostatin analogs, targeted therapies, PRRT, and chemotherapy. In addition, specific medical symptomatic therapies may be used in combination with antitumor therapy to achieve optimal symptomatic control.
